# Persistent DNA Double-Strand Breaks After Repeated Diagnostic CT Scans in Breast Epithelial Cells and Lymphocytes

**DOI:** 10.3389/fonc.2021.634389

**Published:** 2021-04-23

**Authors:** Natalia V. Bogdanova, Nina Jguburia, Dhanya Ramachandran, Nora Nischik, Katharina Konzok, Georg Stamm, Thomas Werncke, Frank Wacker, Thilo Dörk, Hans Christiansen

**Affiliations:** ^1^ Radiation Oncology Research Unit, Hannover Medical School, Hannover, Germany; ^2^ Gynaecology Research Unit, Hannover Medical School, Hannover, Germany; ^3^ Department of Radiology, Hannover Medical School, Hannover, Germany; ^4^ Department of Diagnostic and Interventional Radiology, University Medical Center, Göttingen, Germany

**Keywords:** computed tomography, low-dose x-ray exposure, DNA double-strand breaks, persistent repair foci, radiation-induced long-term response

## Abstract

DNA double-strand break (DSB) induction and repair have been widely studied in radiation therapy (RT); however little is known about the impact of very low exposures from repeated computed tomography (CT) scans for the efficiency of repair. In our current study, DSB repair and kinetics were investigated in side-by-side comparison of RT treatment (2 Gy) with repeated diagnostic CT scans (≤20 mGy) in human breast epithelial cell lines and lymphoblastoid cells harboring different mutations in known DNA damage repair proteins. Immunocytochemical analysis of well known DSB markers *γ*H2AX and 53BP1, within 48 h after each treatment, revealed highly correlated numbers of foci and similar appearance/disappearance profiles. The levels of *γ*H2AX and 53BP1 foci after CT scans were up to 30% of those occurring 0.5 h after 2 Gy irradiation. The DNA damage repair after diagnostic CT scans was monitored and quantitatively assessed by both *γ*H2AX and 53BP1 foci in different cell types. Subsequent diagnostic CT scans in 6 and/or 12 weeks intervals resulted in elevated background levels of repair foci, more pronounced in cells that were prone to genomic instability due to mutations in known regulators of DNA damage response (DDR). The levels of persistent foci remained enhanced for up to 6 months. This “memory effect” may reflect a radiation-induced long-term response of cells after low-dose x-ray exposure.

## Introduction

Computed tomography (CT) and radiotherapy (RT) are currently used for diagnosis and treatment of different diseases, and both rely on ionizing radiation (IR). In regard to known inter-individual variation in radiosensitivity (1), the applicable dose and severity of side effects may be influenced not only by the total dose applied, the dose per fraction, tumor volume and individual cellular radiosensitivity, but also by genetic factors (1, 2). The use of CT has increased over the past decades (3), and recurrent radiological imaging procedure impacts higher cumulative radiation doses to patients than anticipated (4) thereby raising concerns about the possible risks associated with diagnostic ionizing radiation exposure, since evidence has indicated the presence of residual DNA double-strand breaks (DSBs) in human cells exposed to very low radiation doses (5, 6) and probably less efficient repair of DSBs induced by low doses (7). Various factors, including CT parameters which influence the dose in clinical settings (8), and other individual components such as radiosensitivity or capacity for repair, can have an impact on the biological radiation damage in terms of diagnostic and interventional radiology (9).

The cumulative risk of cancer from diagnostic CT scans has been estimated between 1.5 and 2.0% in the United States (10) and ranged from 0.6 to 1.8% in another 13 developed countries (11) with younger persons at the highest risk due to their possibly increased radiosensitivity and longer life expectancy (11–13). These estimates are based on the linear non-threshold model, suggesting that cellular effects occur proportionally to ionizing radiation exposure at all levels; thus no point can be considered risk-free (14). Others have suggested the presence of a damage threshold determining the efficiency of repair (5, 15), especially in terms of the hyper-radiosensitivity phenomenon, which describes radiation survival response of mammalian cells at doses below 0.5 Gy (or at very low doses below 10 cGy) after acute exposure (16).

Ionizing radiation is a well-known genotoxic agent that causes several lesions in affected cells, the most critical being DSBs which can lead to cell death or malignant transformation (17–19). Once DNA gets damaged after x-ray exposure and DSBs form, the histone H2 variant H2AX is phosphorylated at Ser139 through PI3K related kinases, including DNA-PK and the ataxia telangiectasia mutated (ATM) proteins (20, 21). Phosphorylated H2AX, termed *γ*H2AX is required for the assembly of DNA repair proteins at DSB sites and for the activation of checkpoint proteins which arrest the cell cycle progression (22). Another damage sensor, the P53-binding protein 1 (53BP1), accompanies *γ*H2AX at DSBs and signals chromatin damage (23). At the microscopic level, the recruitment of *γ*H2AX or 53BP1 to DSBs leads to the formation of nuclear foci, a phenomenon also described (though less pronounced) for other proteins involved in DNA damage repair or signaling, such as ATM, NBN, RAD50 or MRE11 (23, 24). DSBs lead to *γ*H2AX formation within minutes, and its accumulation *in vitro* shows a linear relationship with the radiation dose over a broad dose range (5, 25), so that the counting of stimuli-induced foci per nucleus in relation to their background levels can be used as a biomarker for DNA damage (5, 26) and their kinetic profiles in biological dosimetry (27, 28). For instance, an association between foci number and absorbed dose has been established *in vitro* after molecular radiotherapy (29) or after short-term partial-body irradiation for CT scans *in vitro* (30, 31), *in vivo* (30, 32) and for patients in radiation oncology including breast cancer (BC) (33, 34). Radiation-induced DSBs evaluated by counting *γ*H2AX and 53BP1 foci as direct responses to radiation and the sensitivity of these assays have provided a basis for the adoption of both these DSB markers as well-established quantitative readouts for DNA targeted treatments in terms of radiation therapy, radionuclide therapy, certain chemotherapies, or combinations thereof (27, 33, 35, 36). However, most of these studies were typically performed at high doses, while only a few addressed the radiation response after low radiation doses (5, 6) or less efficient repair of DSBs, induced by low doses (6, 7).

We have been interested in investigating how radiation-induced repair foci formation is affected by systematic diagnostic CT scans. The main objective of this study was to evaluate the effects of diagnostic chest–abdomen mono-phasic CT on the DSB repair and kinetics through immunocytochemical analysis of *γ*H2AX and 53BP1 foci in a human breast epithelial cell model. A secondary objective was to investigate how radiation-induced repair may vary in repeated diagnostic CT scans by different genetic mutational backgrounds in comparative analyses of breast cancer cell lines and additionally in lymphoid cells with mutations in known regulators of DNA damage repair. We therefore monitored, in parallel, the accumulation and fate of radiation-induced foci within 48 h after conventional radiotherapy treatment and after three rounds of periodic CT scans in order to test the possibility of genetically modulated changes in radiosensitivity after repeated CT exposures.

## Materials and Methods

### Cell Culture

We employed the reference breast epithelial cell line MCF10A as a model for non-malignant breast epithelium. Selected experiments were extended to two triple-negative BC cell lines HCC1395 and HCC1937 and, as an ancillary tissue type, lymphoblastoid cells (LCLs) from a healthy donor and from ataxia-telangiectasia (A-T) patient providing controls with different radiosensitivity phenotypes. Epithelial cell lines were obtained from the American Type Culture Collection (ATCC). MCF10A cells were cultured in MEBM, supplemented with MEGM™ Single Quots™ according to the manufacturer’s instructions (Lonza). Breast cancer epithelial cell lines HCC1395 and HCC1937 were cultured in RPMI 1640 with 10% fetal calf serum, 500 U/ml penicillin, 0.5 mg/ml streptomycin, and 2 mM L-Glutamine. Lymphoblastoid cells HA56 (A-T) and HA325 (healthy donor’s cells) were established *via* transformation of B-lymphocytes from peripheral blood by Epstein–Barr virus (37) and were cultured in RPMI1640 with 15% fetal calf serum and supplements as above. Additionally, for each irradiation setting non-immortalized peripheral blood lymphocytes (PBLs) from one healthy donor were included. PBLs were isolated through Ficoll (GE Healthcare) density-gradient and kept in culture for 3 days in LCL medium. All cells were grown at 37°C in a humidified atmosphere supplemented with 5% CO2. After each CT round one portion of the cells (except in the case of PBLs) were kept and further cultured for 6 weeks (or additionally for 12 weeks in a replication study on MCF10A) in order to undergo subsequent diagnostic CT scans. Cells underwent a total of three rounds of CT with either 6 or 12 weeks (replication experiment on MCF10A) intervals in between each round.

### X-Ray Irradiation *In Vitro*

In order to achieve dose values for the cells which would be comparable to routine staging exams, an Alderson-Rando phantom (Alderson Research Laboratories Inc, Long Island City, New York, USA) was used to simulate a patient of approximately 70 kg. The cell cultures were placed on the chest area of the phantom (**Supplementary Figure 1**). CT scans were performed using a 16-slice Lightspeed scanner (GE Health CareMilwaukee, US). The applied protocol was a mono-phasic CT chest–abdomen scan as used for routine staging examinations. The scan parameters were: tube voltage 120 kV, total collimation 20 mm (16 × 0.6126 mm) with a pitch of 1.375, rotation time 1 s, tube load 170 mAs and noise index 22 for automatic exposure control. The scan length was 30 cm in order to cover the chest. The resulting computed tomography dose index (CTDIvol) was 10.5 +/− 0.4 mGy, and the total dose length product was 360 +/− 13 mGy * cm. The displayed CTDIvol value at the scanner console at the end of the examination is directly correlated with a mean dose value inside the irradiated volume of interest. We did not directly measure the dose in the samples, but employed a well-known and accepted approach to quantify and evaluate real patient dose values using CTDIvol and DLP together with conversion factors. Applying the software CT-Expo we matched the CT scanner and estimated the organ dose for the breast region with a given value of 18 mGy (38). CT scans were repeated every 6 weeks (or 12 weeks in a replication study on MCF10A cells) to simulate the time delay between consecutive staging examinations in a clinical setting. We first employed the time interval of 6 weeks between CT scans, replicating a shortest follow-up period in oncology, which typically ranges from 6 to 12 weeks. In total, we performed three rounds of CT, such that all cell lines had single, double, and triple CT treatments. Untreated values were included in each experimental setting in such a way that for every cell investigated an age-matched control was incorporated. IR at a dose of 2 Gy was applied to all the cell lines using a Mevatron MD-2 accelerator (Siemens, Munich, Germany), under conditions equivalent to the usual application of one fraction for breast cancer radiotherapy. This dose of 2 Gy also served as a positive control for DSB formation since it constitutes the upper end of the linear response range for counting foci (*γ*H2AX) using ICC methods (39).

### Immunocytochemistry

For immunocytochemistry, breast epithelial and BC cells were seeded on cover glasses in sterile non-coated six-well plates two days before treatment in sub-confluent condition. One six-well plate was seeded per condition and time-point. On every day of the next two days fresh medium was added to the cells and cells with more than 80% confluency were treated either with 2Gy or underwent diagnostic CT. Immediately after X-ray application, 1 ml of appropriate fresh medium was added to the cells. At the time of irradiation, adherent cells were immersed in 2 ml medium. Suspensions LCLs and PBLs were also treated in six-well plates, containing 2 ml of appropriate medium and centrifuged at various time points after IR or CT on cover glasses using a Cytospin ROTANTA 460/460R centrifuge (Hettich). All cells were fixed with 3% (w/v) PFA, 2% (w/v) Sucrose in PBS for 10 min and permeabilized with 0.2% (v/v) Triton X-100 in PBS. Cells were incubated simultaneously with antibodies against Phospho (S139)–Histone H2AX (Millipore, clone JBW301) at a ratio of 1:200 and against 53BP1 (Bethyl Laboratories, #A300-272A) at a ratio of 1:400 in 2% (w/v) normal goat serum (NGS, Dianova) for 1 h. After several PBS washing steps, the cells were incubated simultaneously with Alexa Fluor anti-mouse IgG 488 or Alexa Fluor anti-rabbit IgG 546 (Invitrogen, both at a ratio of 1:250) for 45min. The DNA was counterstained with DAPI (Invitrogen), and the cells were mounted with ProLong^®^ Gold (Invitrogen).

### ICC Data Evaluation

For quantitative analyses, foci were counted by two independent trained observers using a Leica DMI6000B microscope with 63× objective and a 1.6× magnification. One observer was blinded to the nature of the samples. In order to detect foci in all three dimensions, the observer manually focused on each z-stack throughout the nucleus. The counting process for suspension cells was performed independently in several (up to five) different areas of slide until at least 50 “positive” cells (with foci) per position were detected and registered. In adherent cells, the counting process was performed until a minimum of 1,000 cells were registered. Every responsive cell (with one or more repair foci) was included in the evaluation. Of note, in the replication study on MCF10A, freshly used cell cultures exhibited somewhat higher basal levels of foci. Cells were counted independent of the cell-cycle phase, but cells with apoptotic morphology or cells with an intensely stained nucleus were excluded from the counting process. For PBLs, monocytes and granulocytes were excluded from the analysis according to the morphological criteria. For counting of foci in LCLs and PBLs, an Olympus FV1000 confocal microscope was used to overcome layer problem by visualization. For the replication study, automated foci quantification was also performed (to test for any observer bias). Automated counting procedure was applied using “LAS X 2D Analysis Multi Channel Extension” licensed software module for quantitative microscopy. The results from manual and automated counting approaches exhibited very high similarity and were not statistically different (**Supplementary Figure 2**).

### Cell Proliferation and Senescence-Associated Beta-Galactosidase Activity

Cell proliferation was measured by 5-ethynyl-2′-deoxyuridine (EdU) incorporation into newly synthesized DNA and its recognition by azide dyes *via* a copper mediated “click” reaction, using Click-iT^®^ EdU Imaging Kit (Invitrogen). Briefly, cells were seeded on cover glasses in sterile non-coated six-well plates in sub-confluent condition and incubated with 10 mM of EdU for 4 h. The cells were then fixed with 3.7% paraformaldehyde; EdU detection was carried out according to the supplier’s instructions, and nuclei were stained with Hoechst 33342 for the following analysis. For the detection of cells with replicating DNA, Alexa Fluor^®^ 488 labeled cells were counted with a Leica DMI6000B microscope using a 20× objective and 1.6× magnification. The counting process was performed independently in several (up to five) different areas of the slide until at least 500 cells per slide were detected and registered.

SA-*ß*-gal staining was performed using the staining kit (Cell Signalling Technology) to detect the pH-specific (pH 6.0) activity of *β*-galactosidase, which is associated with senescence (40). The procedure was followed according to the manufacturer’s instructions. Briefly, sub-confluent cells were seeded either in 12-well plate or cover glasses (for additional staining procedures afterwards), and the development of blue color was documented 24 h after the fixation and staining procedure. To interfere with replication fork progression and induce replicative stress associated with a senescence-like state, 0.5 mM hydroxyurea treatment for 96 h was performed prior to fixation as a control for *β*-galactosidase activity in MCF10A cells (41). Pictures of 12-well plates with the staining solution remaining on the cells were taken using Nikon Eclipse TS100 inverse microscope. Quantification was performed using Image J software. The number of senescent cells was normalized to the total cell number counted (up to 1,000 cells per well). In parallel to EdU incorporation and SA-*β*-gal activity measurement, cells were additionally stained for 53BP1 (as above). Briefly, cells treated with SA-*β*-gal solution were permeabilized (0.5% v/v Triton X-100 in PBS) and blocked with 2% NGS for 20 min; whereas cells treated with EdU prior to nuclei staining were blocked with 2% NGS for 20 min. Both were incubated with antibodies against 53BP1 and Alexa Fluor anti-rabbit IgG 546; DNA was counterstained either with DAPI or Hoechst 33342, respectively. The cells were then mounted with ProLong^®^ Gold.

### Statistical Analysis

Formation and resolution of foci within 48 h after each treatment was statistically analyzed using GraphPad Prism (version 9.0.0; Graphpad Software). 1% False discovery rate (FDR) was used to identify and eliminate outliers from each dataset using the ROUT method. In order to compare differences between two groups, a student’s t-test was performed. Three or more groups were compared using one-way ANOVA (a repeated-measures analysis of variance), and a linear trend test was performed for multiple comparison between consecutive groups where indicated. P values at α < 0.05 were considered significant. Irradiation experiments in the main run were performed in biological triplicates (for some values also technical replicates are included). In the replication study, biological duplicates were analyzed. Data are presented as bar plots with average foci number (+/− SEM) per cell. “Aged” untreated estimates were not statistically different from untreated estimates before the first CT (student’s t-test, data not shown), thus those values were pooled together as untreated (“UNT”) for more convenient graphical presentation. Pearson correlation coefficients (r) were calculated between the average number of *γ*H2AX and 53BP1 foci.

## Results

### *γ*H2AX and 53BP1 Foci in MCF10A Breast Epithelial Cells

**Figure 1:**
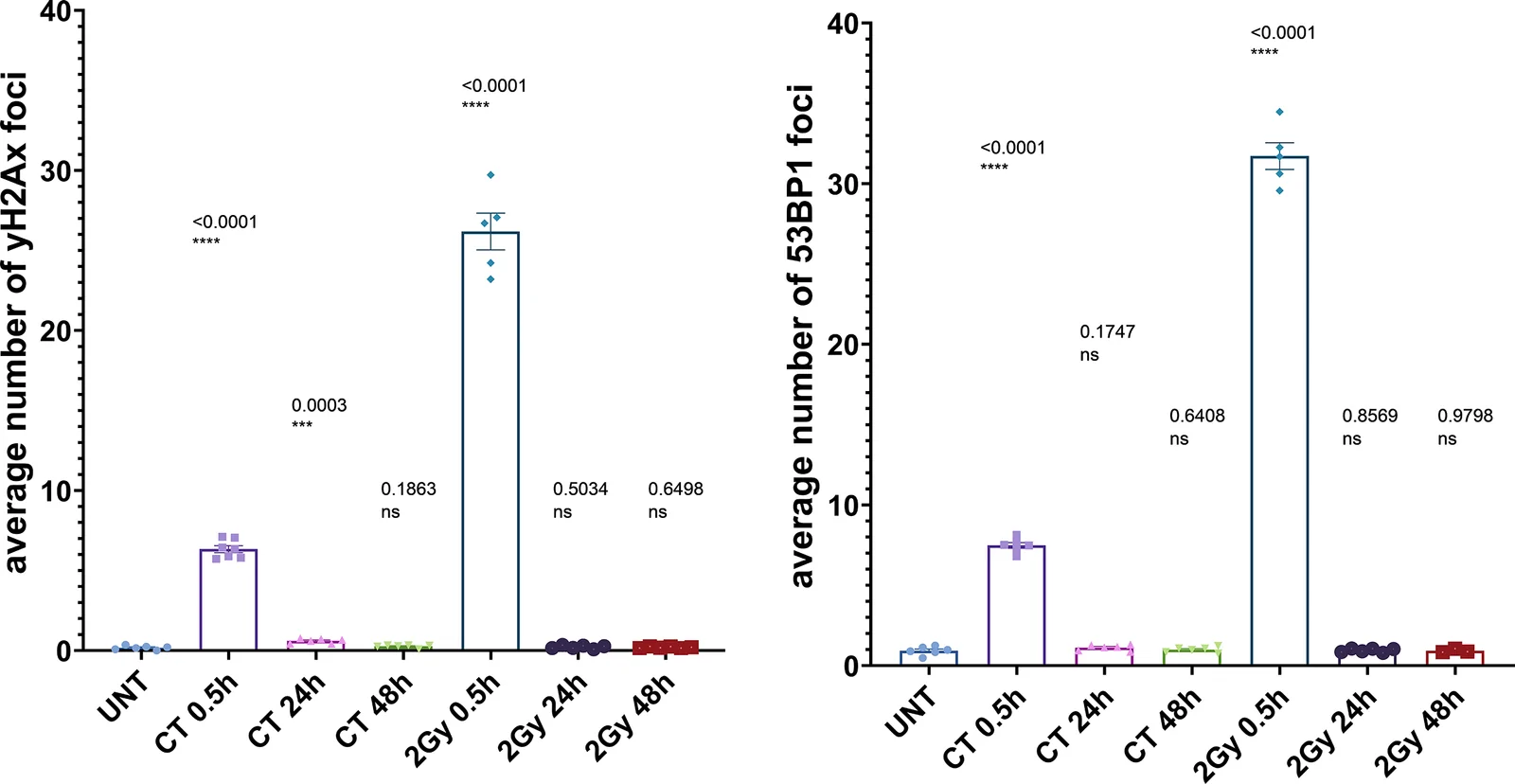
Immunocytochemical analysis of repair foci after single dose in MCF10A cells. Evaluation of *γ*H2AX foci (left) or 53BP1 foci (right) after irradiation with either 20 mGy (CT) or 2 Gy, respectively; using conventional fluorescence microscopy (Leica DMI6000B). Data are presented as bar plots with the average foci number (+/− SEM) per cell per experiment from at least three independent experiments (UNT, untreated values with included “age-matched” controls; p values on graph represent comparison to UNT; ns, not significant; SEM, standard error of the mean). ***P≤0.001, ****P<0.0001.

**Figure 2:**
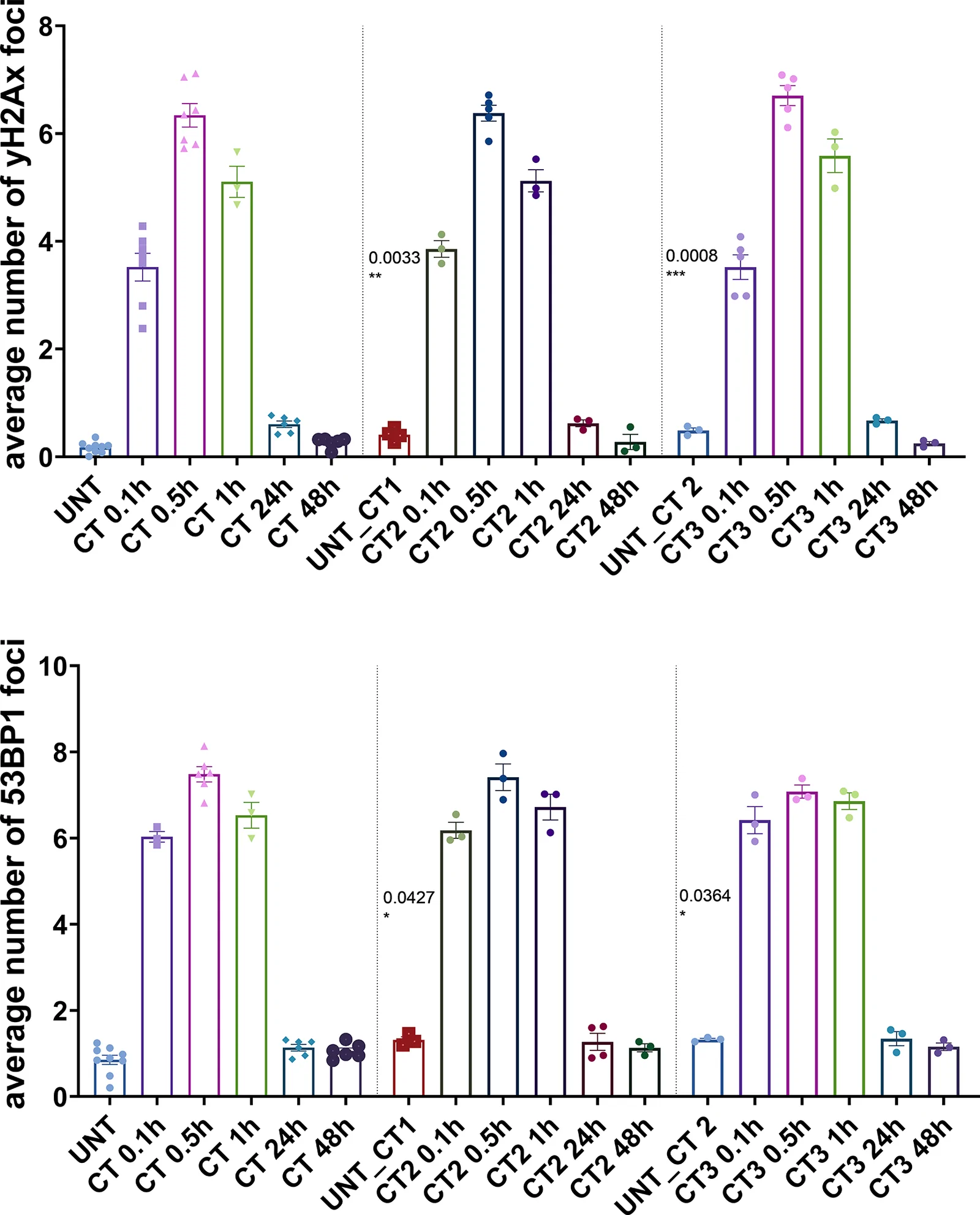
Immunocytochemical analysis of repair foci after repeated CT scans in MCF10A cells. Evaluation of *γ*H2AX foci (top) and 53BP1 foci (bottom) after systematic diagnostic CT scans with ~20 mGy per round (in total three rounds) using conventional fluorescence microscopy (Leica DMI6000B). Data are presented as bar plots of average foci number (+/− SEM) per cell per experiment from at least three independent experiments (UNT, untreated values with included “age-matched” controls; CT1, 1st round of computed tomography; CT2, second subsequent diagnostic CT; CT3, third subsequent diagnostic CT; p values on graph represent comparison to UNT; SEM, standard error of the mean). *P≤0.05 , **P≤0.01, ***P≤0.001.

**Figure 3:**
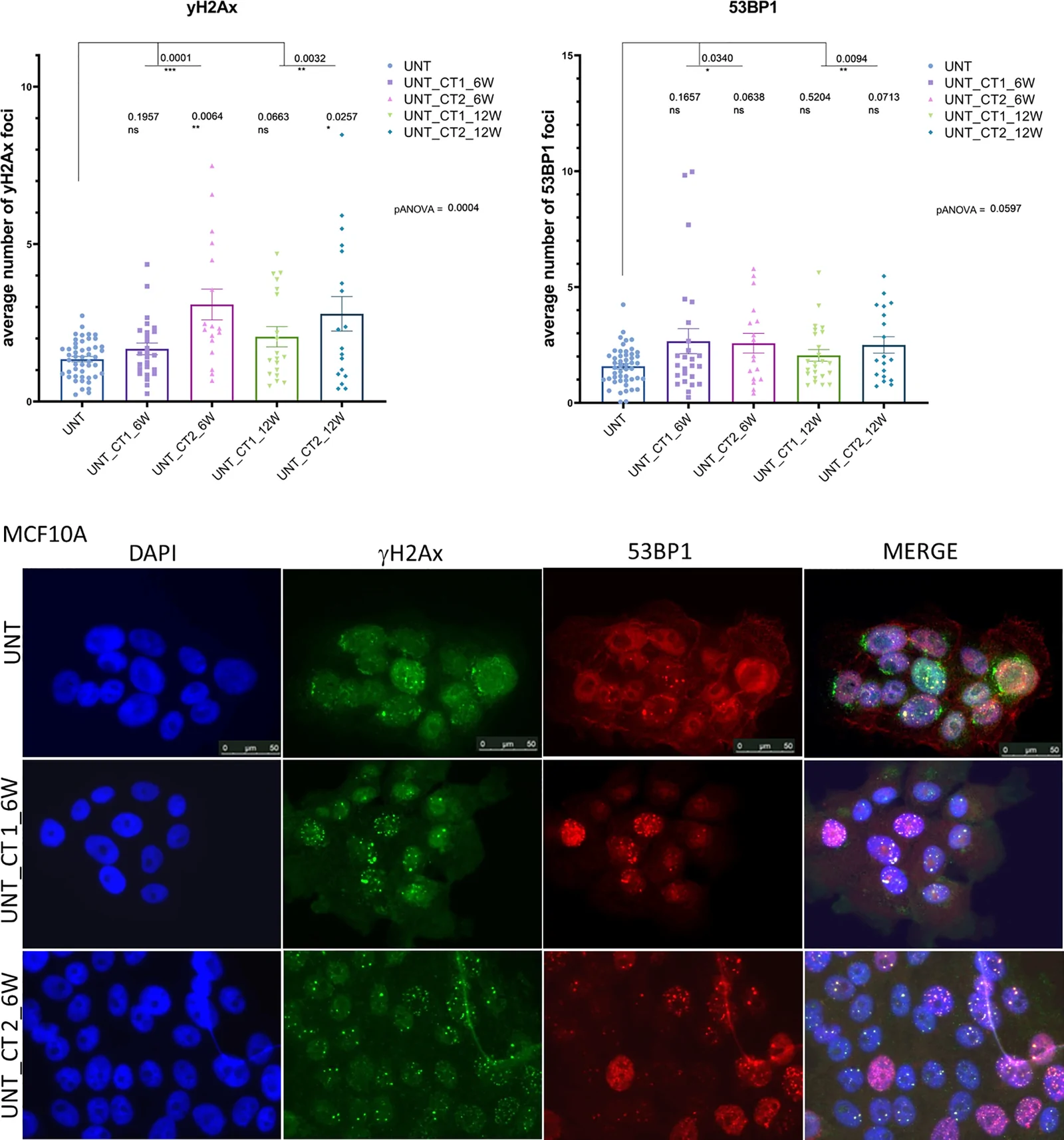
Immunocytochemical analysis of background persisting foci number after repeated CT scans in MCF10A cells (replication study). Upper Panel: Evaluation of *γ*H2AX foci (left) and 53P1 foci (right) from repeated experimental settings in 6 and 12 weeks intervals after systematic diagnostic CT scans with 20 mGy per round (in total three rounds) using conventional fluorescence microscopy (Leica DMI6000B). Data are presented as bar plots of average number of foci (+/− SEM) per cell. Each data point represents randomized counting area of slide from two independent experiments (biological replicates) and two technical replicates (UNT, untreated values with included “age-matched” controls; CT 1, cells, which went once through the CT and were further cultured for 6 or 12 weeks, respectively; CT 2, cells, which went twice through the CT after 6 or 12 weeks; ns, not significant; SEM, standard error of the mean). Lower panel: Example of H2AX foci (green) and 53BP1 foci (red) double immunostaining. DNA is counterstained with DAPI (UNT, untreated value “age-matched” to UNT_CT 2; UNT_CT 1, cells were cultured 6 weeks after the 1st round of computed tomography; UNT_CT 2, cells were further cultured for 6 weeks after the second subsequent diagnostic CT and went through two CT rounds). *P≤0.05 , **P≤0.01, ***P≤0.001.

To investigate the DDR in our cell culture model, we first monitored *γ*H2AX and 53BP1 foci formation in MCF10A cells up to 48 h after exposure to x-rays (2 Gy and single CT application). For CT implementation we utilized a specifically developed phantom for a precise estimation of a clinically comparable dose to be applied to cell cultures. As expected for irradiation with 2 Gy, the average numbers of *γ*H2AX and 53BP1 foci were highest at 0.5 h after treatment (**Supplementary Table 1**) with a notable decrease (similar to untreated cells) within 48 h after irradiation (**Figure 1**) with almost 100% of foci removal for *γ*H2AX and 53BP1 respectively (**Supplementary Table 2**). After a single dose of ≤20 mGy for the CT treatment, a comparable to the 2 Gy experiment trend was observed with a 0.5 h peak and similar time course (**Figure 1** and **Supplementary Tables 1**, **2**). MCF10A cells showed significantly induced levels of *γ*H2AX and 53BP1 foci at 0.5 h after CT treatment, with a reduction at 24 h. At 48 h after stimuli, the foci numbers further decreased and were almost comparable to untreated levels for *γ*H2AX (p = 0.19) (**Figure 1** and **Supplementary Tables 1**, **2**) with nearly 90% of foci removal for *γ*H2AX, although levels for 53BP1 with about 25% of residual foci (**Supplementary Table 2**) were somewhat elevated. We then examined whether the course of DDR would be altered if cells were treated by multiple rounds of CT. At first, the reference cell line, MCF10A, was examined in three rounds of subsequent CT scans with intervals of 6 weeks. After each CT round, the number of foci was evaluated at 0.1, 0.5, 1, 24, and 48 h after treatment (**Figure 2**). MCF10A cells showed significantly elevated levels of *γ*H2AX and 53BP1 foci after each CT treatment, with maximum at 0.5 h in every round (p < 0.0001 for both types of foci), with a reduction at 24 h (around 80–90% for both foci types) and further decrease at 48 h with similar time course over all three rounds of CT (**Figure 2**, **Supplementary Tables 1**, **2**). However, cells which had undergone CT exhibited an elevated number of persisting background foci at the beginning of the next round of the CT, suggesting an accumulation of DNA damage (**Figures 2** and **3** upper panel, **Supplementary Table 3**). This difference was notably significant for *γ*H2AX foci in each individual round (CT1: p < 0.003 and CT2: p < 0.001) and less significant for 53BP1 foci (CT1: p < 0.05 and CT2: p < 0.04). Moreover, from the second CT round on, the remaining foci levels were apparently elevated in contrast to the untreated state (**Supplementary Table 3**). Overall, the CT treatment evoked about 20–25% the level of *γ*H2AX and 53BP1 foci compared with the 2 Gy treatment. It thus seemed that 2 Gy dose was more efficient in the foci induction (higher foci appearance rate at 0.5 h), whereas foci from CT application were slower in their disappearance rate (**Figure 1**, **Supplementary Tables 1**, **2**, **4**) up to 48 h after stimuli. There was a highly significant correlation between *γ*H2AX and 53BP1 foci (**Supplementary Figure 3**, right panel). Subsequently we repeated the experiment for MCF10A cells with fresh cell cultures used. The analysis was restricted to three time-points (0, 0.5, and 48 h). Cells were additionally subjected to prolonged intervals of 12 weeks between CT scans, and elevated numbers of persisting foci were again seen after each round of CT with 6 and/or 12 weeks intervals in the replication study (**Figure 3A**, upper panel). The difference between untreated cells and pre-treated cells at CT2 was significant for *γ*H2AX foci after both 6 and 12 weeks intervals, whereas, for 53BP1 foci, it was only nominally significant (**Figure 3**, upper panel). Both automated and manual procedures of foci counting were not statistically different (**Supplementary Figure 2**).

### *γ*H2AX and 53BP1 Foci in Breast Cancer Cell Lines

We then tested whether these observations can also be extended to commonly used breast cancer cell models that have gathered DDR deficiencies. Therefore, HCC1395 and HCC1937 TNBC cell lines were additionally investigated. Both cell lines are *BRCA1*-mutant BC lines with HCC1395 carrying an additional mutation in *NBN* that impairs *γ*H2AX accumulation (42) among other mutations that possibly could modify the DDR response. As expected for irradiation with 2 Gy, there were clear differences between the cell lines with different mutational backgrounds, especially in contrast to the reference MCF10A cells (**Supplementary Figure 4**, **Supplementary Tables 1**, **2**, **5**). However, HCC1395 had a higher ratio of 53BP1/H2AX foci which was consistent with its known NBN deficiency (42). After CT application, a trend comparable to the 2 Gy experiment was observed with a 0.5 h peak and similar time course for both breast cancer cell lines exposed to a single dose of ≤20 mGy (**Supplementary Figure 4**, **Supplementary Tables 1** **, 2**). As expected, HCC1395 cells displayed reduced yield of both γH2AX and 53BP1 foci in comparison to MCF10A at 0.5h after CT (**Supplementary Table 5**), but these foci remained elevated at 24h (p = 0.01 for γH2AX and p=0.03 for 53BP1, respectively) and 48h (p = 0.04 for γH2AX and p=0.01 for 53BP1 foci,) after stimuli in comparison to the untreated condition (**Supplementary Figure 4**). The HCC1937 cell line exhibited an increase in *γ*H2AX and 53BP1 foci at 0.5 h after CT and also showed increased residual levels of foci at 24 h after treatment (p < 0.001 for both types of foci in comparison to the untreated condition (**Supplementary Figure 4**, **Supplementary Tables 1**, **2**). Similar to MCF10A, *γ*H2AX and 53BP1 foci numbers in comparison to the untreated condition were reduced at 48 h after CT (p = 0.52 for *γ*H2AX and p = 0.20 for 53BP1 foci) in HCC1937 cells. Overall, the CT treatment evoked about 30–35% the level of *γ*H2AX and 53BP1 foci compared with the 2 Gy treatment (**Supplementary Table 1**). Similar to MCF10A, relative to the untreated state, foci levels remained elevated 48 h after CT application with some increment across the rounds for both cell lines (**Supplementary Table 3**), more prominently in HCC1395. There was a highly significant correlation between induced *γ*H2AX and 53BP1 foci for both cell lines (**Supplementary Figure 3**, right panel). The higher ratio of 53BP1/*γ*H2AX foci in HCC1395 observed with 2 Gy was similarly observed with diagnostic CT scans. Additionally, an analysis of our BC cell lines restricted to three time-points (0, 0.5, and 48 h) showed similar trends towards elevated background levels of foci after subsequent CT treatments (**Supplementary Figure 5**, **Supplementary Table 3**). This difference was significant in HCC1395 and HCC1937 cells after each round of CT for *γ*H2AX (CT1: p < 0.05 and p < 0.05, respectively, CT2: p < 0.001 and p < 0.05, respectively), and for 53BP1 some trend was observed in the first CT round for HCC1395 (CT1 p = 0.07) and in the second round for HCC1937 (CT2 p = 0.05), respectively.

### *γ*H2AX and 53BP1 Foci After Single-Dose Irradiation And Multiple CT Treatments in Lymphoblastoid Cells and Lymphocytes

We tested and confirmed the robustness of our findings in lymphoblastoid cells, including one LCL from an A-T patient expected to display a radiosensitivity phenotype. Foci in the LCLs were evaluated and counted using confocal microscopy. After 2 Gy IR treatment, the ATM-deficient LCL (HA56) showed significant differences in *γ*H2AX and 53BP1 foci induction at 0.5h when compared to the wild-type control line (HA325), as well as a significantly elevated number of residual *γ*H2AX and 53BP1 foci up to 48 h (**Supplementary Figure 6** upper panel, **Supplementary Tables 1**, **2**, **5**). Upon CT treatment, the A-T cells were able to accumulate *γ*H2AX and 53BP1 foci to a similar extent as the control LCL at 0.5h after irradiation (**Supplementary Table 1**) but showed evidence for an attenuated repair at 24 h; however, the number of *γ*H2AX and 53BP1 foci remained significantly increased until 48h post treatment (**Supplementary Figure 6**, upper panel, **Supplementary Table 2**). Upon the multiple CT treatment both LCLs exhibited similar foci kinetics as by single-dose (**Supplementary Figure 7**). The trend with elevated background levels of *γ*H2AX and 53BP1 foci in successive CT rounds was visible for LCLs HA325 and HA56 (**Supplementary Figure 7**, **Supplementary Table 3**) as well, though with statistical significance only in HA56 cells (*γ*H2AX: p = 0.003 for CT1 and p = 0.0003 for CT2, respectively; and 53BP1: p = 0.044 for CT1 and p = 0.047 for CT2, respectively). Foci levels in contrast to the untreated state remained elevated 48 h after CT application with some increment across the rounds for LCLs, especially in A-T cells (**Supplementary Table 3**). This was consistent for both markers which again were highly correlated in the LCLs (**Supplementary Figure 3**, left panel). We additionally included native PBLs for each experimental condition to verify that the CT effects can be observed through quantitative evaluation of *γ*H2AX and 53BP1 foci in primary lymphocytes. PBLs behaved in a similar manner to wild-type LCLs in 53BP1 monitoring, though the average yield of *γ*H2AX foci appeared somewhat lower after CT treatment (**Supplementary Figure 6** lower panel, **Supplementary Tables 1**, **2**, **4**). Overall, the CT treatment evoked around 20% (in lymphocytes) and ˜30% (in lymphoblastoid cells) of *γ*H2AX and 53BP1 foci compared to the 2 Gy treatment (**Supplementary Table 1**), which was similar to the effect seen in breast epithelial cells. *γ*H2AX and 53BP1 foci from CT application showed slower resolution in PBLs 24 h after stimuli (**Supplementary Figure 6** lower panel, **Supplementary Tables 2**, **4**).

### Persisting DSBs and Senescence in MCF10A Cells

**Figure 4:**
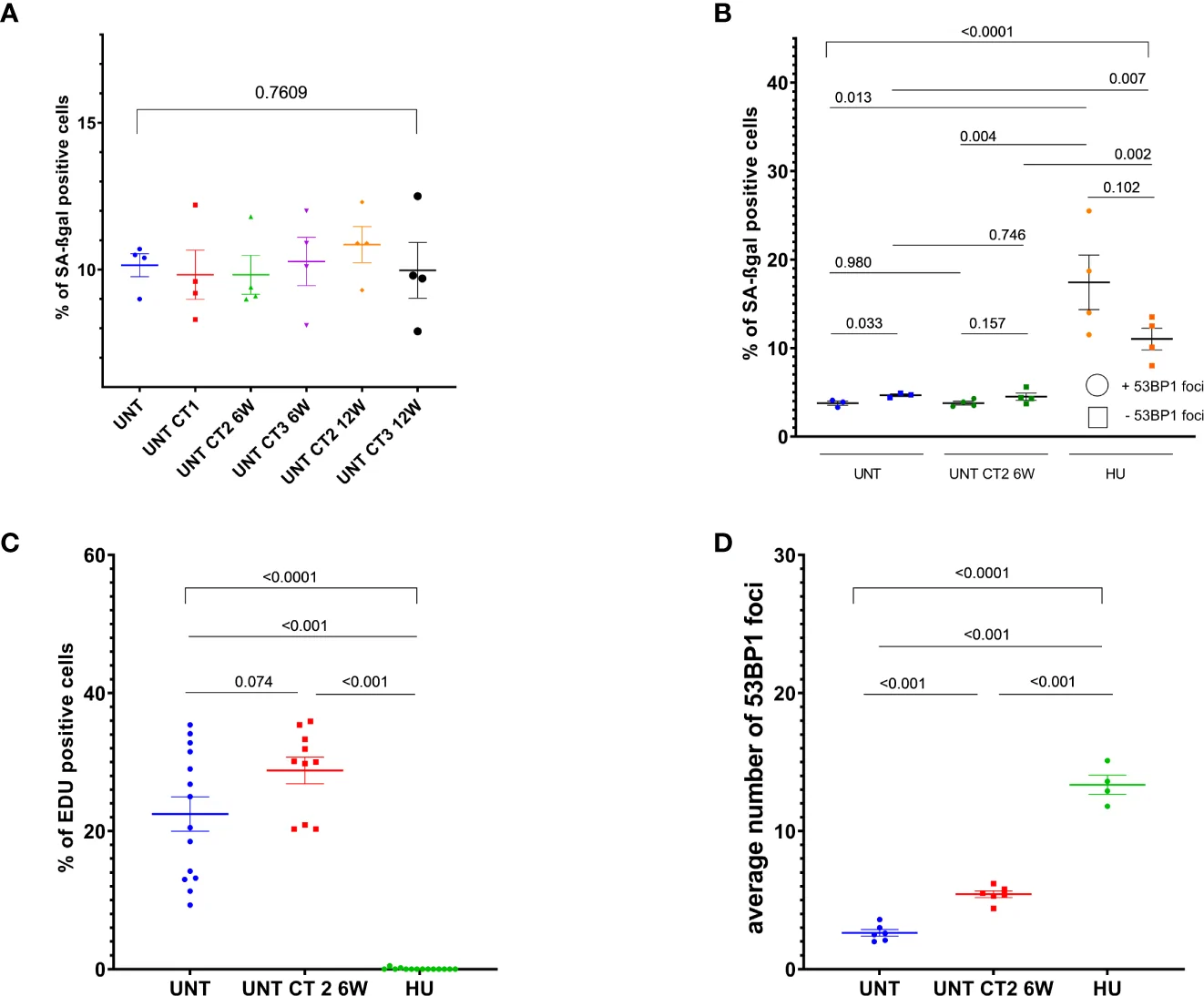
Cell proliferation and senescence-associated beta-galactosidase (SA-*β*gal) activity analysis in MCF10A cells. Evaluation of SA-*β*gal staining **(A, B)** and EdU incorporation **(C, D)** in cells, which went through diagnostic CT scans and were further cultured for 6 weeks, using either inverse microscopy (Nikon Eclipse TS100)—**(A)**, or conventional fluorescence microscopy (Leica DMI6000B) — **(B–D)**. Data are presented as bar plots +/− SEM. **(A)** Percentage of SA-*β*gal positive cells in untreated (12 weeks “aged”) and pre-treated at different CT rounds MCF10A cells in 6 and 12 weeks intervals. **(B)** Percentage of SA-*β*gal positive cells with or without 53BP1 foci. **(C)** Percentage of EdU positive cells. **(D)** Average 53BP1 foci number per cell, assessed after EdU labeling. Data from two independent experiments includes one technical duplicate. Each dot in **(C)** represents counting area (UNT, untreated age-matched to UNT_CT2_6 weeks control; CT1, cells after the 1st round of computed tomography; CT2, cells after the second subsequent diagnostic CT, HU, 0.5 mM hydroxyurea treatment for 96 h prior to fixation in MCF10A cells as a control for senescence-like state and replicative stress; SEM, standard error of the mean).

We tested whether higher background levels of persistent foci after subsequent CT treatments are the cause of senescence or aging in the long-term cell culture, and therefore measured *β*-galactosidase activity and EdU incorporation in MCF10A cells which had undergone diagnostic CT scans and were further cultured for 6 or 12 weeks intervals between subsequent CT rounds. Senescence was assessed in MCF10A cells by means of *β*-galactosidase activity. We observed no elevation in the numbers of senescent cells and no reduction in EdU incorporation after diagnostic CT scans along with elevated number of persisting 53BP1 background foci (**Figure 4** and **Supplementary Figure 8**). Furthermore, cells showing SA-*ß*-gal activity did not show more 53BP1 foci in comparison to *ß*-gal negative cells as assessed by ICC (**Supplementary Figure 8**). These results excluded senescence or proliferative exhaustion as the main mechanism behind the accumulation of foci after repeated CT scans. EdU incorporation analysis, along with 53BP1 foci staining and 53BP1 foci evaluation after *β*-galactosidase stain, was performed in age-matched untreated and pre-treated at CT2 MCF10A cells after 6 weeks intervals, since the difference for this time point was significant in the main and following experiment (**Figures 2**, **3**).

## Discussion

Ionizing radiation induces an extensive DSB repair that helps cells to survive radiation toxicity and avoid subsequent chromosomal translocations (17, 18). While the response towards radiation in higher dose ranges, typically used for radiation therapy, has been well characterized, there is still a need to elucidate the consequences of low-dose ionizing radiation typically used in diagnostic applications such as computed tomography. Approximately 14% of the total annual exposure to IR is caused by diagnostic x-ray procedures (10). CT provides the largest input to medical radiation doses (4, 43) with the risk of possible additional DNA damage. Furthermore, modern, often expensive oncologic therapies require regular follow-up studies for therapy response assessment and restaging. Consecutive CT studies with intervals between 6 and 12 weeks are the accepted standard in patient care. However, due to more expensive and faster acting anti-cancer drugs, there is a trend towards shorter follow-up cycles to assess early response. Hence the risk of low dose radiation exposure is a topic of intense and sometimes controversial discussions, emphasizing the necessity of studies investigating the effects of low radiation doses (44, 45), since evidence is accumulating that risk estimates based on the LNT (linear-no-threshold) model may potentially underestimate the risks of CT procedures.

In the present study the experimental setup utilized a standard CT used in our hospital. Ionizing radiation was applied to the cell cultures embedded in an Alderson phantom, thus realistically simulating daily clinical practice. To assess the effects of the ionizing radiation, we monitored the accumulation of the two repair proteins *γ*H2AX and 53BP1 at DNA DSBs, both of which are well-established quantitative readouts for chromosome breaks in radiation therapy (26, 27, 33, 34). However, they are not yet commonly used to evaluate diagnostic CT scans, albeit *γ*H2AX foci analysis by immunofluorescence represents a very sensitive method for detection of DSBs after irradiation in smaller doses such as 1–2 mGy (5, 6). Our data are in line with some results from a previous study (46), in which DNA damage was induced by a single cardiac CT in blood samples that also were placed within a phantom. A correlation between the physical exposure parameters and *γ*H2AX was reported. Our present work has compared breast epithelial cells and lymphoid cells, additionally investigated the effect on 53BP1 foci, and, most notably, further analyzed the effect of repeated CT scans with defined intervals. Our study is, to the best of our knowledge, also the first to provide a side-by-side comparison of a radiation therapy treatment (2 Gy) with repeated computed tomography scans (≤20 mGy) in their effects under clinical conditions that are routinely applied to patients.

Several studies reported on the induction of *γ*H2AX foci by x-ray CT exposure in adult and pediatric patients and association with elevated DNA damage levels (8, 30–32, 47). Our data are in line with these published studies. We assessed elevated levels of foci per cell, corresponding to induced DSBs, with a maximum seen at 30 min after exposure. The disappearance rate of *γ*H2AX foci, which reflects the completion of the repair process (5, 48), has been previously shown to be associated with sensitivity to x-rays (32, 35), and *γ*H2AX foci analysis has been proposed for radiosensitivity screening in terms of clinically relevant doses of radiation (32, 34, 35) or to determine dose-related effects on IR induced DSBs levels (5, 8). Of note, the kinetics for the loss of *γ*H2AX foci has been shown to depend on the radiation dose applied, and cellular response to DSBs was found to be substantially different for low *versus* high radiation doses with slower repair after doses in the mGy range (5, 6, 49).

The levels of *γ*H2AX and 53BP1 foci after CT scans were up to 30–60% of those occurring after 2 Gy irradiation with similar appearance/disappearance profiles. The kinetic profiles in our experimental settings were highly similar for both *γ*H2Ax and 53BP1 proteins. Our correlation analysis shows that 53BP1 foci can be used largely equivalently to *γ*H2AX foci. Both are sensitive biomarkers for low doses commonly applied in CT and can be reliably assessed also in lymphocytes as shown in the current study, although their use is limited due to the fact that this cell line harbors. In one previous study, both *γ*H2AX and 53BP1 have been investigated in radioiodine based therapy setting, and the authors found that both markers were similar in foci number, suggesting that both proteins are useful markers for detecting radiation exposure after radionuclide incorporation, even for absorbed doses in the blood below 20 mGy (27). This is largely confirmed in our study, although we also report one epithelial cell line in which *γ*H2AX foci are underrepresented in comparison to 53BP1 foci. This is possibly due to the fact that this line harbors a mutation in the NBN protein that is known to interact with H2AX and thereby selectively triggers its formation of extended foci (42). In a more recent study, 53BP1 has been also suggested as a more sensitive marker for the evaluation of induced DNA repair foci in human lymphocytes (28). Our findings are partially in line with these observations, since we monitored a lower yield of *γ*H2AX foci in PBLs after CT irradiation. Further, 53BP1 foci from CT application appeared somewhat “slower” in resolution 24 h after CT application, possibly due to a higher technical sensitivity at low doses. It is interesting to note, that all investigated cells showed some “slower” rate of foci loss after CT treatment.

We have further observed that background levels of *γ*H2AX foci and 53BP1 foci were elevated in comparison to the “age-matched” untreated cells after each following CT round in 6 and 12 weeks intervals in our reference breast epithelial cell line MCF10A. This observation was then reproduced and found to be particularly pronounced in 6 weeks interval analysis in cells that were impaired in DNA double-strand break repair, harboring mutations in *BRCA1* (HCC1395, HCC1937) and *NBN* (HCC1395), respectively. The double mutant HCC1395 line showed the most pronounced response. Further work would be needed to determine whether these differences seen were in fact due to mutation in *BRCA1* and/or *NBN*, since additional somatic events during long-term culturing, which might have impacted on DDR response in these cells, could not be excluded. However, similarities in elevated background levels of repair foci were also observed in ATM deficient lymphoblastoid cells known to be radiosensitive. Thus, residual DSBs induced by repeated CT scans seemed to accumulate especially in radiosensitive cells, while they were seemingly more efficiently repaired by wild-type cells. Such damage accumulation in the mutant cells might translate to cellular radiosensitivity even at low doses. All investigated cells showed significant contrasts of the second and third rounds of CT to the first CT round, although irradiation responses in each CT round behaved as independent acute insults, reflecting some adaptation mechanism. The kinetics of strand break rejoining was found to be not influenced by adaptation to irradiation in previous experiments with low dose (50) and therapeutic doses of x-ray (51). Induction of *γ*H2AX foci was found to be affected by the initial radiation exposure with a smaller number of foci induced by subsequent exposures in both studies, but research from Mariotti et al. (51) reported a recovery time of 12 h for full induction of *γ*H2AX foci upon the next insult. Our observations extend these findings, insofar that in our settings cells were challenged with subsequent irradiation after 6 weeks and showed similar foci induction as after single dose application. Regarding the increase in background levels of repair foci, we cannot exclude the formation of some *de novo* DSBs as a result of cell metabolism, which is a steady process in every cell. However, without assuming a “memory effect”, this likely had to occur to the same extent in untreated and pre-treated cells, being a natural phenomenon. In our analysis of “age-matched” untreated cells and cells pre-treated with CT, a significant difference in numbers of foci per cell was found, suggesting some other mechanisms than *de novo* formed DSB or temporary lesions. It is noteworthy that these foci seem to persist for more than 6 months. Over the past years, a number of studies reported a small but significant number of focal DDR signals persistent in irradiated cells, which were termed ‘unrepairable DSBs’ (52–54). However, these studies were typically performed at high radiation doses, with only a few addressing the radiation response after low exposure, and the effects were largely assessed after only one application of radiation or total observation time was no longer than 24 h (28, 52–55).

Unrepairable foci which persisted for a minimum of 70 days have been described in normal human skin diploid fibroblasts after 6 Gy irradiation (55). The authors further found that cultured irradiated cells, after an additional challenge with x-rays, were competent in repairing newly generated foci, similar to the foci resolution kinetics after only an initial dose. However, newly arisen breaks formed additional unrepairable DSBs, which then accumulate. These foci may be distinct from our observations with regard to dependence on radiation doses and growth conditions, since Noda and co-authors observed the formation of unrepairable DSB foci at high dose and in non-replicating, irradiated cells. The authors also observed induction of premature senescence along with formation of the unrepairable foci. This close association between the formation of radiation-inducible unrepairable DSBs and senescence has been also described by others (53, 55–59). The results from our study, with regard to the low doses used, do not indicate that unrepairable foci in our experimental settings are the cause of senescence, nor do they appear to occur due to accumulation of rare, spontaneous DNA damage during long-term cell culture (**Figure 4** and **Supplementary Figure 8**). Moreover, in regard to mitotic catastrophe after irradiation, which in low dose ranges is linked to a so-called low-dose hypersensitivity phenomenon, and is related as a long-term outcome to senescence, one recent study described an experimental in silico model showing that in the case of DNA repair accommodation after a low-dose radiation, survival rate is higher and mitotic catastrophe index is lower (60). It has been also demonstrated by others that persistent *γ*H2AX and 53BP1 foci do not block cell proliferation, being compatible with cell-cycle progression and transmission into daughter cells after high-dose (5 Gy) irradiation (61), or were induced by low dose (80 mGy). This may allow, in principle, a long-term persistence of residual foci. Alternatively, a more frequent formation of *de novo* foci could be hypothesized, perhaps due to pre-sensitized DSB signaling pathways. Residual *γ*Н2АХ foci were predominantly observed in the proliferating cells and do not play a role in delayed irradiation consequences associated with cellular senescence (62).

Residual, unrepaired DSB foci have been reported in cells that were treated for mild replication stress (63, 64) or were exposed to very low IR doses (5, 6). The authors found that chromosome breaks on top of the persistent level of ~0.1 *γ*H2Ax foci per cell do not lead to the accumulation of DSBs through an efficient repair, whereas repair at/or below this level is strongly compromised (5), and the kinetics of *γ*H2AX foci loss in confluent cells is substantially compromised after doses of 10 mGy and lower (6). An effect of longer persisting residual foci at low doses ≤10 cGy than at a higher dose was further described in human lymphocytes (28). Along with this, the authors report that effects of low doses can be, in principal, extrapolated from higher doses using *γ*H2AX residual foci and proposed both *γ*H2AX and 53BP1 as very useful markers for low dose biodosimetry *in vitro*. This is an important issue for radiation protection and prediction of possible health effects. Thus, the observation of persistent DSB repair foci may also be relevant for cancer risk inference. Studies of either the induced or persistent DNA damage foci have reported some predictive value to estimate subsequent cancer risk (31, 65). These parameters are not equivalent: Foci at 0.5 h are markers of intact DSB signaling while persistent foci are markers of inefficient DSB repair. Both defective signaling and defective DNA repair can give rise to cancer so that measures at different time-points after treatment should be most informative. Persistent foci may thus be taken as markers of cancer risk. If no residual foci are found, it needs to be taken into account whether DNA damage signaling was in the normal range since chromosome breaks may otherwise have gone undetected, and the cancer risk would be nevertheless increased.

The structures containing persisting DSBs, their role and consequences are still unclear and largely a matter of debate. Several mechanisms could be discussed for persisting *γ*H2AX foci, as, for instance delayed or ineffective *γ*H2AX de-phosphorylation or *γ*H2AX removal from the chromatin (66, 67). However, our results are seen for 53BP1 foci as well. It is also worth to notice bystander effects, the mechanism of which is implicated in the hypersensitivity phenomenon and contributes to adaptive response and is not cell cycle-based. The production of such “secondary” DNA DSBs in bystander cells (68) may also impact on levels of persistent foci. Considering the complexity of the cellular response to ionizing radiation and common knowledge gaps in some aspects (especially in low dose ranges), one could hypothesize that DSBs induced by ≤20 mGy low doses x-rays of CT scans in our experimental settings can remain unrepaired and lead to persisting foci. It is possible that such residual foci just mark unrepairable damage or they may serve to “prime” the cell at particularly vulnerable sites for a more efficient DNA damage response upon the next insult (adaptive response). Although radiation exposure induces DSBs randomly in the chromosome, DNA lesions that resist cell repair activities and became persistent in the form of genuine DSBs (69) are proposed to occur proximal to telomeres or mark unrepairable telomeric DNA (53, 70), or possibly remain as unrepairable DSBs inside the chromosome (54). Bystander or secondary foci can be also generated by transcriptional activity (71), indicating perhaps the existence of some transcriptional program in “primed” cells, which results in modification of chromatin structure with broken DNA ends, possibly protecting the DNA from genomic catastrophe and may be transmitted to progeny. More recently, a role of damage-induced non-coding RNAs in determining breakpoint recognition in heterochromatic DNA was revealed (72). It will be interesting to see which alterations in damage sensing and repair activities are associated with this form of long-term “memory” of the DNA damage.

Apart from the novel insights obtained in the present study, some limitations should be acknowledged. First, the utilization of established cell lines could have partially affected some results and cannot fully recapitulate the *in vivo* situation. This is due to the need of immortalization which affects cell cycle regulation and genomic stability. However, our study design included long-term (up to six months) observations for which primary cell cultures could not be employed. Second, only two cell types were investigated, and although our observations were largely consistent between them, DNA repair capacity might vary between tissues and more models might provide an even more comprehensive evaluation of DSB dynamics in systematic diagnostic CT scans.

## Conclusions

We have shown that a DDR can be reliably monitored and quantitatively determined in breast epithelial cells, lymphoblastoid cells, and peripheral lymphocytes exposed to diagnostic x-ray at doses below 20 mGy. Our findings indicate that *γ*H2AX or 53BP1 foci are largely equivalent biomarkers for the assessment of DSB repair capacity, which is crucial for estimating the response to radiation exposure. In the clinical perspective, such foci analyses may prove to be valuable tools to determine individual radiosensitivity during the diagnostic process, and automation will facilitate and improve the screening of larger cohorts with potentially valuable impact on the individualization of diagnosis and treatment. Importantly, our observations indicate that repeated diagnostic CT scans can result in elevated levels of background *γ*H2AX and 53BP1 foci that persist over a longer period of time. This outcome was notably evident in both cells with and without genomic instability but it seemed to be higher in those with genomic instability. This kind of “memory effect” may reflect a radiation-induced long-term response of cells after low-dose x-ray exposure. Further studies will be necessary to elucidate the mechanisms behind these observations.

## Data Availability Statement

The raw data supporting the conclusions of this article will be made available by the authors, upon reasonable request.

## Author Contributions

NB, FW, TD, HC: study concepts. GS, TW: study design. NJ, NN: data acquisition. NB, KK, DR: quality control of data. GS, TW: quality control of dosimetry. NJ, NN, KK: data analysis. DR, TD, NB: statistical analysis. NB and TD: data interpretation. NB, NJ, DR, KK: manuscript preparation. TD, DR, HC: manuscript editing. NB, TD, FW: manuscript review. All authors contributed to the article and approved the submitted version.

## Conflict of Interest

The authors declare that the research was conducted in the absence of any commercial or financial relationships that could be construed as a potential conflict of interest.

## Supplementary Material

Supplementary Figure 1 Parameters of CT examination settings. Typical exposure protocol with dose report (left panel). Volume rendering of the Alderson-Rando phantom with the applied cell cultures (right panel).

Supplementary Figure 2 Comparison of manual and automated (software based) procedures for foci counting. γH2AX foci (left) or 53BP1 foci (right) scored with either manual (M) or automated method (LAS), respectively. Conventional fluorescence microscopy (Leica DMI6000B) “per eye” and “LASX 2D Analysis Multi Channel Extension” licensed software module for quantitative microscopy for automated technique were applied. Exemplary results from replication study on MCF10A in 6 weeks intervals after systematic diagnostic CT scans are presented as bar plots with the average foci number (+/− SEM) per cell. Each data point represents one counting area on slide from two independent experiments (biological replicates) and two technical replicates. (CT, cells, which went once through the CT and were further cultured for 6 weeks, respectively; CT 2, cells, which went twice through the CT after 6 weeks; SEM, standard error of the mean; p values generated using student’s t-test).

Supplementary Figure 3 Correlation analysis of CT-induced repair foci. The correlation between average *γ*H2AX and 53BP1 foci numbers in cell lines was tested after systematic diagnostic CT scans, shown here as scatter plots. Left panel represents LCLs: wild-type control (HA325) and A-T cell line (HA56). Right panel shows reference breast epithelial cell line MCF10A and BC cell lines HCC1395, HCC1937. X-axis: mean number of 53BP1 foci; Y-axis: mean number of *γ*H2AX foci.

Supplementary Figure 4 Immunocytochemical analysis of repair foci after single dose in BC cell lines. Evaluation of *γ*H2AX foci (top) or 53BP1 foci (bottom) after irradiation with either ˜20 mGy (CT) or 2 Gy, respectively, using conventional fluorescence microscopy (Leica DMI6000B). Data are presented as bar plots with the average foci number (+/− SEM) per cell per experiment from at least three independent experiments (HCC1395, BC cell line, double-mutant in *BRCA1/NBN* and HCC1937, BC cell line with *BRCA1* mutation, UNT, untreated values with included “age-matched” controls; p values on graph represent comparison to UNT; ns, not significant; SEM, standard error of the mean). *P≤0.05 , **P≤0.01, ***P<0.0001.

Supplementary Figure 5 Immunocytochemical analysis of repair foci after repeated CT in BC cell lines. Evaluation of *γ*H2AX foci (top) and 53BP1 foci (bottom) after systematic diagnostic CT scans with ˜20 mGy (in total three rounds) using conventional fluorescence microscopy (Leica DMI6000B). Data are presented as bar plots of average foci number (+/− SEM) per cell per experiment from at least three independent experiments (UNT, untreated values with included “age-matched” controls; CT1, 1st round of computed tomography; CT2, second subsequent diagnostic CT; CT3, third subsequent diagnostic CT; ns, not significant; SEM, standard error of the mean). *P≤0.05 , **P≤0.01.

Supplementary Figure 6 Immunocytochemical analysis of repair foci after single dose in lymphoid cells. Evaluation of *γ*H2AX foci (left) or 53BP1 foci (right) after irradiation with either 20 mGy (CT) or 2 Gy, respectively, using confocal microscopy (Olympus FV1000). Upper panel: wild-type and A-T LCLs, lower panel: PBLs from healthy donor. Data are presented as bar plots of average foci number (+/− SEM) per cell per experiment from at least three independent experiments (HA325, wild-type LCL, HA56, A-T LCL. Lower panel—PBLs from healthy donor. UNT, untreated values with included “age-matched” controls; p values represent comparison to UNT; ns, not significant; SEM, standard error of the mean). *P≤0.01, **P≤0.001, ***P<0.0001.

Supplementary Figure 7 Immunocytochemical analysis of repair foci after repeated CT in lymphoblastoid cells. Evaluation of *γ*H2AX foci (top) and 53P1 foci (bottom) after systematic diagnostic CT scans with ˜20 mGy (in total three rounds) using confocal microscopy (Olympus FV1000). Data are presented as bar plots of average number of foci (+/− SEM) per cell per experiment from at least three independent experiments (UNT, untreated values with “age matched” controls, CT, 1st round of computed tomography, CT2, second subsequent diagnostic CT, CT3, third subsequent diagnostic CT; ns, not significant; SEM, standard error of the mean). *P≤0.05 , **P≤0.01, ***P≤0.001.

Supplementary Figure 8 Cellular senescence-associated beta-galactosidase (SA-*β*gal) activity and cell proliferation in untreated and pre-treated in CT scans MCF10A cells. **(A)** SA-*β*gal activity in cells, went through diagnostic CT scans and further cultured for 6 and 12 weeks, using inverse microscopy Nikon Eclipse TS100 (UNT, untreated “age matched” to UNT_CT2_6 weeks; CT1, 1st round of computed tomography; CT2, second subsequent diagnostic CT; CT3, third subsequent diagnostic CT; HU, 0.5 mM hydroxyurea treatment for 96 h prior to fixation as a control for senescence-like state and replicative stress. **(B)** Average 53BP1 foci number per cell, assessed after SA-*β*gal staining, using conventional fluorescence microscopy Leica DMI6000B. 53BP1 foci numbers were evaluated in SA-*β*gal+, SA-*β*gal—cells, in total minimum 100 cells per condition were counted. Data are presented as bar plots +/− SEM (UNT, 12 weeks “aged” untreated; CT2, second round of diagnostic CT; HU, 0.5 mM hydroxyurea treatment; SEM, standard error of the mean). **(C)** Example of 53BP1 foci (red) immunofluorescence labeling after senescence-associated SA-*β*gal staining. DNA is counterstained with DAPI and (D) example of 53BP1 foci (red) immunostaining after EdU incorporation (green). DNA is counterstained with Hoechst (UNT, untreated “age matched” to UNT_CT2_6 weeks; UNT_CT 2, cells were cultured in 6 weeks intervals after two rounds of CT, HU, 0.5 mM hydroxyurea treatment).
