# Supplementary figures and images for: Persistent DNA Double-Strand Breaks After Repeated Diagnostic CT Scans in Breast Epithelial Cells and Lymphocytes
Source: Front Oncol. 2021 Apr 23;11:634389. doi: 10.3389/fonc.2021.634389 (PMC8103218; doi:10.3389/fonc.2021.634389)

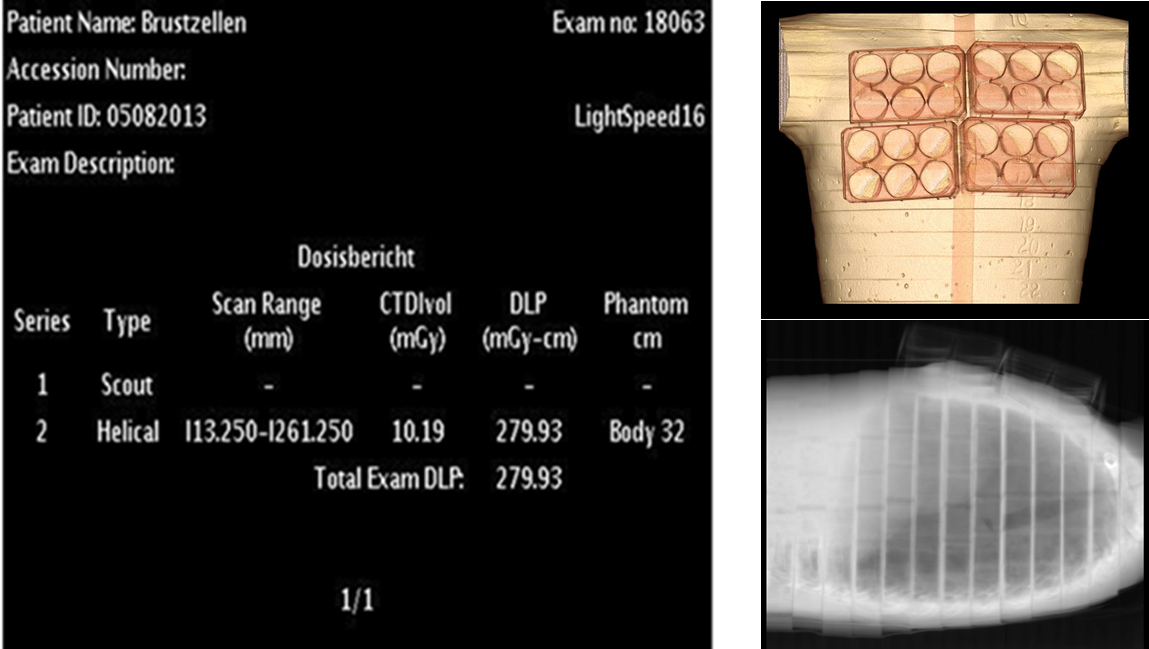

Supplement: Supplementary Figure 1 — Parameters of CT examination settings. Typical exposure protocol with dose report (left panel). Volume rendering of the Alderson-Rando phantom with the applied cell cultures (right panel). [file Image_1.tif]

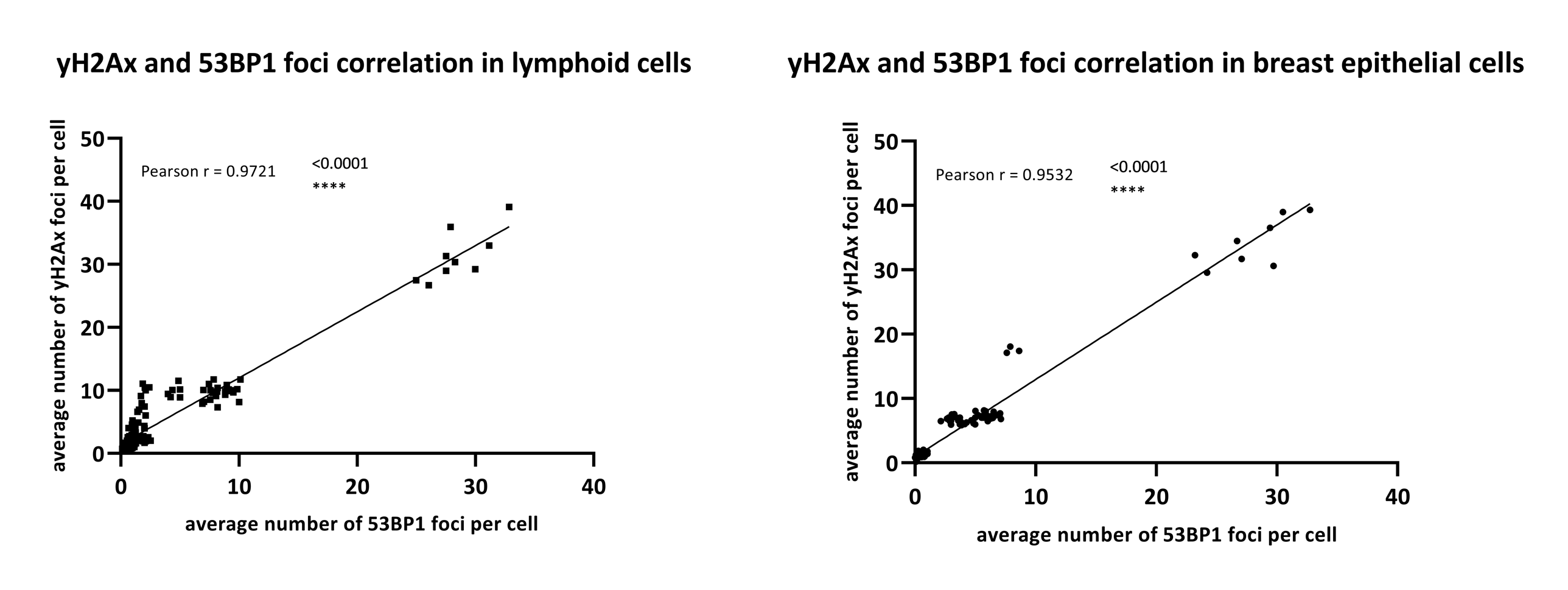

Supplement: Supplementary Figure 3 — Correlation analysis of CT-induced repair foci. The correlation between average γH2AX and 53BP1 foci numbers in cell lines was tested after systematic diagnostic CT scans, shown here as scatter plots. Left panel represents LCLs: wild-type control (HA325) and A-T cell line (HA56). Right panel shows reference breast epithelial cell line MCF10A and BC cell lines HCC1395, HCC1937. X-axis: mean number of 53BP1 foci; Y-axis: mean number of γH2AX foci. [file Image_3.tif]

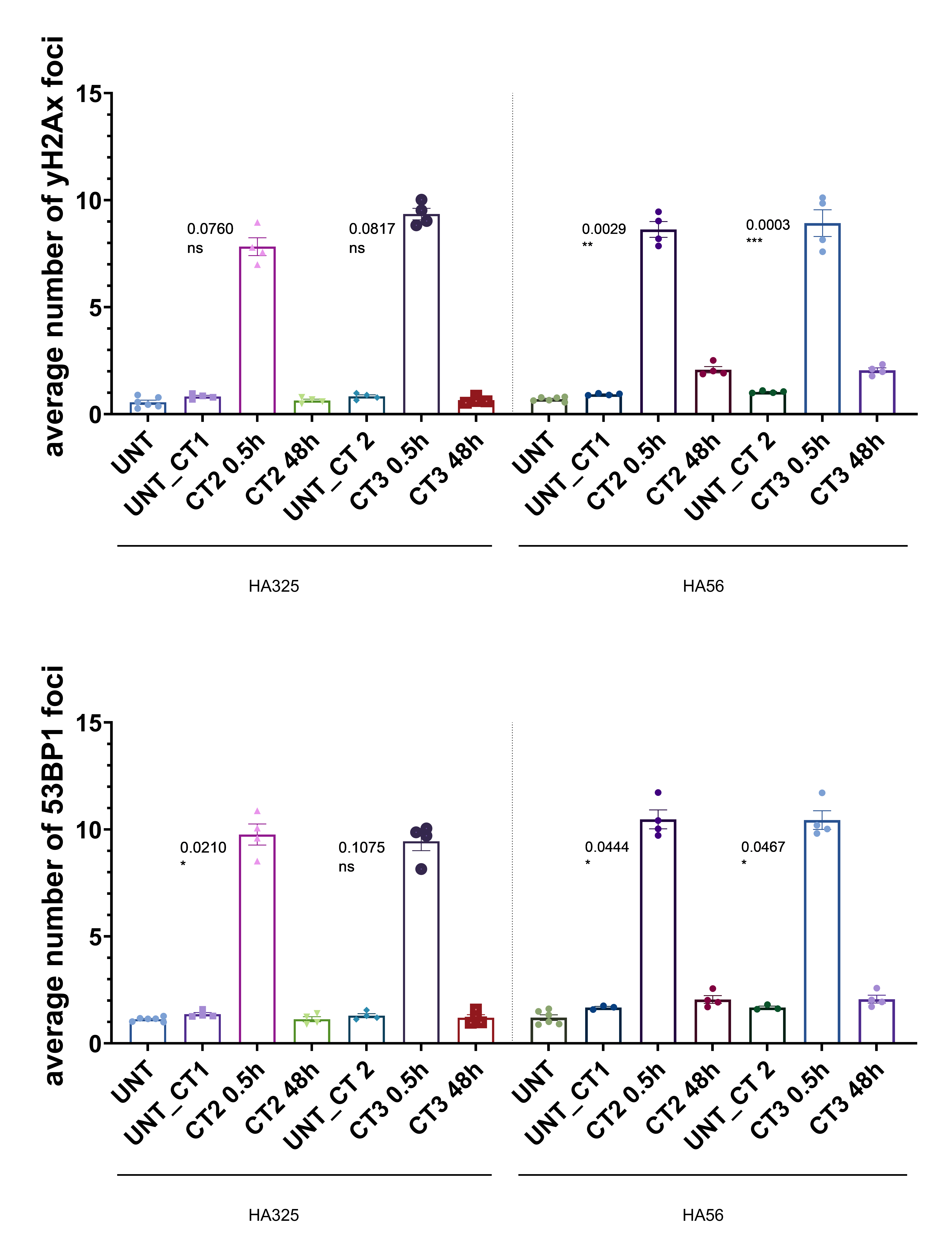

Supplement: Supplementary Figure 7 — Immunocytochemical analysis of repair foci after repeated CT in lymphoblastoid cells. Evaluation of γH2AX foci (top) and 53P1 foci (bottom) after systematic diagnostic CT scans with ˜20 mGy (in total three rounds) using confocal microscopy (Olympus FV1000). Data are presented as bar plots of average number of foci (+/− SEM) per cell per experiment from at least three independent experiments (UNT, untreated values with “age matched” controls, CT, 1st round of computed tomography, CT2, second subsequent diagnostic CT, CT3, third subsequent diagnostic CT; ns, not significant; SEM, standard error of the mean). *P≤0.05 , **P≤0.01, ***P≤0.001. [file Image_7.tif]

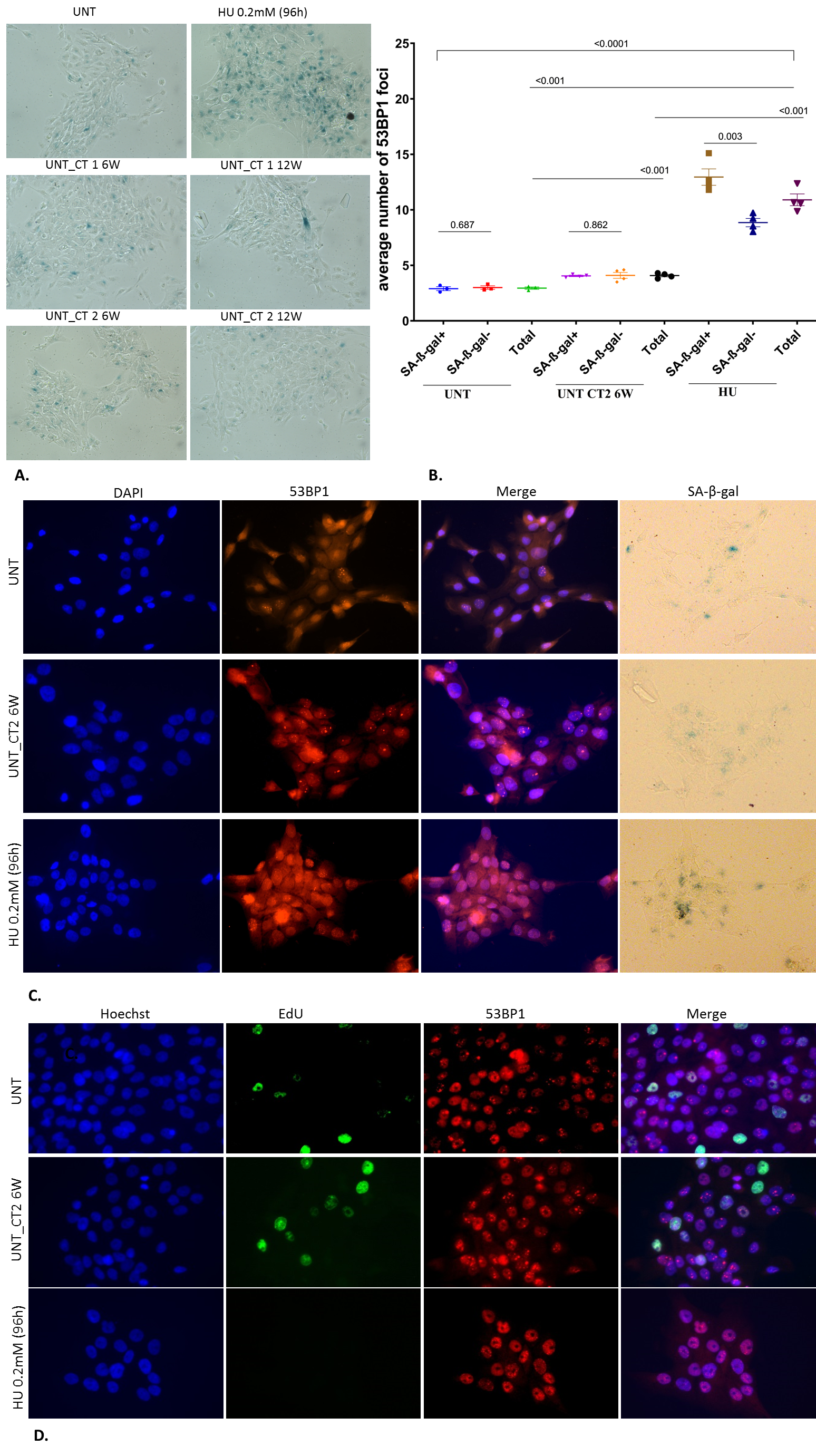

Supplement: Supplementary Figure 8 — Cellular senescence-associated beta-galactosidase (SA-βgal) activity and cell proliferation in untreated and pre-treated in CT scans MCF10A cells. (A) SA-βgal activity in cells, went through diagnostic CT scans and further cultured for 6 and 12 weeks, using inverse microscopy Nikon Eclipse TS100 (UNT, untreated “age matched” to UNT_CT2_6 weeks; CT1, 1st round of computed tomography; CT2, second subsequent diagnostic CT; CT3, third subsequent diagnostic CT; HU, 0.5 mM hydroxyurea treatment for 96 h prior to fixation as a control for senescence-like state and replicative stress. (B) Average 53BP1 foci number per cell, assessed after SA-βgal staining, using conventional fluorescence microscopy Leica DMI6000B. 53BP1 foci numbers were evaluated in SA-βgal+, SA-βgal—cells, in total minimum 100 cells per condition were counted. Data are presented as bar plots +/− SEM (UNT, 12 weeks “aged” untreated; CT2, second round of diagnostic CT; HU, 0.5 mM hydroxyurea treatment; SEM, standard error of the mean). (C) Example of 53BP1 foci (red) immunofluorescence labeling after senescence-associated SA-βgal staining. DNA is counterstained with DAPI and (D) example of 53BP1 foci (red) immunostaining after EdU incorporation (green). DNA is counterstained with Hoechst (UNT, untreated “age matched” to UNT_CT2_6 weeks; UNT_CT 2, cells were cultured in 6 weeks intervals after two rounds of CT, HU, 0.5 mM hydroxyurea treatment). [file Image_8.tif]
