# Supplementary material for: Persistent DNA Double-Strand Breaks After Repeated Diagnostic CT Scans in Breast Epithelial Cells and Lymphocytes
Source: Front Oncol. 2021 Apr 23;11:634389. doi: 10.3389/fonc.2021.634389 (PMC8103218; doi:10.3389/fonc.2021.634389)
Supplement: Supplementary file 9 [file Table_1.doc]

**Supplementary Table 1**. Mean values of DDR foci induction in percent ( ± standard error of the mean) after 2Gy and CT application.

| **treatment** | **foci type** | **MCF10A** | **HCC1395** | **HCC1937** | **HA325** | **HA56** | **PBLs** |
| --- | --- | --- | --- | --- | --- | --- | --- |
| **2Gy_0.5h** | ***γH2Ax*** | 1,5x10^4^  ±9,8 | 4,3x10^3^  ±6,5 | 5,2x10^3^  ±5,5 | 5,1x10^3^  ±5,6 | 4,3x10^3^  ±3,5 | 1,2x10^4^  ±5,0 |
|  | ***53BP1*** | 3,2x10^3^  ±5,8 | 1,5x10^3^  ±2,8 | 3,2x10^3^  ±4,0 | 2,7x10^3^  ±6,7 | 3,0x10^3^  ±8,5 | 3,6x10^3^  ±4,4 |
| **CT 1_0.5h** | ***γH2Ax*** | 3,3x10^3^  ±16,4 | 1,4x10^3^  ±16,4 | 994,3±10,2 | 1,4x10^3^  ±8,9 | 1,2x10^3^  ±7,0 | 2,0x10^3^  ±9,9 |
|  | ***53BP1*** | 807,0±5,8 | 587,1±7,3 | 600,6±4,4 | 809,7±12,1 | 838,7±8,1 | 1,3x10^3^  ±10,0 |
| **CT 2_0.5h** | ***γH2Ax*** | 3,7x10^3^  ±5,2 | 1,6x10^3^  ±8,4 | 975,6±3,2 | 1,4x10^3^  ±10,6 | 1,3x10^3^  ±8,5 | n.a |
|  | ***53BP1*** | 870,8±7,2 | 573,0±4,7 | 637,8±6,1 | 867,9±10,1 | 870,5±8,5 | n.a |
| **CT 3_0.5h** | ***γH2Ax*** | 3,9x10^3^  ±6,1 | 1,7x10^3^  ±8,4 | 976,4±10,9 | 1,7x10^3^  ±5,7 | 1,3x10^3^  ±14,0 | n.a |
|  | ***53BP1*** | 831,7±3,7 | 595,7±5,0 | 657,1±5,2 | 839,6±9,3 | 867,6±8,3 | n.a |

n.a – not applicable, CT1– 1st round of computed tomography, CT2– second subsequent diagnostic CT, CT3 – third subsequent diagnostic CT.
