# Supplementary material for: Persistent DNA Double-Strand Breaks After Repeated Diagnostic CT Scans in Breast Epithelial Cells and Lymphocytes
Source: Front Oncol. 2021 Apr 23;11:634389. doi: 10.3389/fonc.2021.634389 (PMC8103218; doi:10.3389/fonc.2021.634389)
Supplement: Supplementary file 10 [file Table_2.doc]

**Supplementary Table 2**. Mean values of DDR foci decay in percent (± standard error of the mean) after 2Gy and CT application.

| **treatment** | **foci type** | **MCF10A** | **HCC1395** | **HCC1937** | **HA325** | **HA56** | **PBLs** |
| --- | --- | --- | --- | --- | --- | --- | --- |
| **2Gy_24h** | ***γH2Ax*** | 99,2±9,5 | 92,9±3,5 | 97,0±5,3 | 92,8±5,1 | 67,5±1,5 | 98,4±4,8 |
|  | ***53BP1*** | 97,0±5,6 | 90,7±0,7 | 95,7±3,9 | 94,3±6,0 | 80,6±9,1 | 96,2±4,3 |
| **2Gy_48h** | ***γH2Ax*** | 99,2±9,7 | 94,4±5,9 | 97,8±4,3 | 97,2±5,0 | 89,9±1,1 | 99,3±4,9 |
|  | ***53BP1*** | 97,1±5,6 | 91,8±2,3 | 96,3±3,9 | 96,3±6,0 | 89,8±7,2 | 97,4±4,0 |
| **CT 1_24h** | ***γH2Ax*** | 89,6±15,5 | 78,8±2,8 | 86,1±9,0 | 81,2±3,5 | 46,2±5,1 | 89,4±7,8 |
|  | ***53BP1*** | 85,1±4,1 | 78,6±7,3 | 77,4±3,8 | 80,3±8,9 | 51,6±4,0 | 85,2±8,7 |
| **CT 1_48h** | ***γH2Ax*** | 95,4±15,6 | 85,1±7,5 | 87,8±9,1 | 91,5±8,2 | 73,9±5,5 | 94,7±8,8 |
|  | ***53BP1*** | 86,8±4,1 | 77,9±7,1 | 81,2±3,8 | 85,3±8,5 | 73,8±2,1 | 92,2±10,0 |
| **CT 2_24h** | ***γH2Ax*** | 90,3±3,8 | n.a | n.a | n.a | n.a | n.a |
|  | ***53BP1*** | 83,0±2,3 | n.a | n.a | n.a | n.a | n.a |
| **CT 2_48h** | ***γH2Ax*** | 95,7±1,5 | 88,1±7,3 | 87,4±1,0 | 91,9±9,8 | 75,9±6,8 | n.a |
|  | ***53BP1*** | 84,7±6,0 | 78,7±1,5 | 81,1±2,4 | 88,5±8,7 | 80,4±6,2 | n.a |
| **CT 3_24h** | ***γH2Ax*** | 90,0±5,9 | n.a | n.a | n.a | n.a | n.a |
|  | ***53BP1*** | 81,0±0,3 | n.a | n.a | n.a | n.a | n.a |
| **CT 3_48h** | ***γH2Ax*** | 96,3±5,5 | 89,2±2,0 | 86,8±9,3 | 93,0±4,5 | 77,0±14,8 | n.a |
|  | ***53BP1*** | 83,7±1,9 | 79,7±1,8 | 81,0±1,5 | 87,4±7,1 | 80,2±6,0 | n.a |

n.a – not applicable, CT1– 1st round of computed tomography, CT2– second subsequent diagnostic CT, CT3 – third subsequent diagnostic CT.
