# Supplementary material for: Persistent DNA Double-Strand Breaks After Repeated Diagnostic CT Scans in Breast Epithelial Cells and Lymphocytes
Source: Front Oncol. 2021 Apr 23;11:634389. doi: 10.3389/fonc.2021.634389 (PMC8103218; doi:10.3389/fonc.2021.634389)
Supplement: Supplementary file 11 [file Table_3.doc]

**Supplementary Table 3.** Linear trend test for CT treatment within the cell lines (One-way ANOVA)

| **Cell line** | **foci type** | **UNT vs UNT_CT1 vs UNT_CT2*** | **CT vs CT2 vs CT3** | |
| --- | --- | --- | --- | --- |
|  |  |  | **0.5h** | **48h** |
| MCF10A | γH2Ax | 0,0002 | 0,2201 | 0,2385 |
|  | 53BP1 | 0,0111 | 0,88 | 0,3908 |
| HCC1395 | γH2Ax | 0,001 | 0,0288 | 0,7989 |
|  | 53BP1 | 0,129 | 0,6841 | 0,0668 |
| HCC1937 | γH2Ax | 0,0208 | 0,7658 | 0,6925 |
|  | 53BP1 | 0,1098 | 0,6754 | 0,5042 |
| HA325 | γH2Ax | 0,0377 | 0,0048 | 0,8758 |
|  | 53BP1 | 0,0753 | 0,5125 | 0,584 |
| HA56 | γH2Ax | <0,0001 | 0,2031 | 0,7466 |
|  | 53BP1 | 0,0153 | 0,5657 | 0,0918 |

*UNT_CT1 – cells which were treated once, UNT_CT2 – cells which were treated twice, age-matched controls are included; CT– 1st round of computed tomography, CT2– second subsequent diagnostic CT, CT3 – third subsequent diagnostic CT.
