# Supplementary material for: Persistent DNA Double-Strand Breaks After Repeated Diagnostic CT Scans in Breast Epithelial Cells and Lymphocytes
Source: Front Oncol. 2021 Apr 23;11:634389. doi: 10.3389/fonc.2021.634389 (PMC8103218; doi:10.3389/fonc.2021.634389)
Supplement: Supplementary file 12 [file Table_4.doc]

**Supplementary Table 4**. Comparison of CT treatment (single dose) and 2Gy within the time points and cell lines (student’s t-test).

| **Cell line** | **CT 0.5h vs 2Gy 0.5h** | | **CT 24h vs 2Gy 24h** | | **CT 48h vs 2Gy 48h** | |
| --- | --- | --- | --- | --- | --- | --- |
|  | ***γH2Ax*** | ***53BP1*** | ***γH2Ax*** | ***53BP1*** | ***γH2Ax*** | ***53BP1*** |
| MCF10A | <0,0001 | <0,0001 | 0,0004 | 0,0792 | 0,1598 | 0,571 |
| HCC1395 | <0,0001 | <0,0001 | 0,8738 | 0,8148 | 0,5956 | 0,3307 |
| HCC1937 | <0,0001 | <0,0001 | 0,3155 | 0,6951 | 0,8961 | 0,3896 |
| HA325 | <0,0001 | <0,0001 | 0,02 | 0,8958 | 0,2533 | 0,4671 |
| HA56 | <0,0001 | <0,0001 | <0,0001 | 0,0147 | 0,0441 | 0,0904 |
| PBLs | <0,0001 | <0,0001 | 0,2889 | 0,0044 | 0,2238 | 0,7318 |
