# Supplementary material for: Persistent DNA Double-Strand Breaks After Repeated Diagnostic CT Scans in Breast Epithelial Cells and Lymphocytes
Source: Front Oncol. 2021 Apr 23;11:634389. doi: 10.3389/fonc.2021.634389 (PMC8103218; doi:10.3389/fonc.2021.634389)
Supplement: Supplementary file 13 [file Table_5.doc]

**Supplementary Table 5.** Comparison of treatments and time points to reference within the cell type (student’s t-test).

| **Treatment and time** | **HCC1395 vs MCF10A** | | **HCC1937 vs MCF10A** | | **HA56 vs HA325** | |
| --- | --- | --- | --- | --- | --- | --- |
|  | ***γH2Ax*** | ***53BP1*** | ***γH2Ax*** | ***53BP1*** | ***γH2Ax*** | ***53BP1*** |
| UNT | 0,8172 | 0,0601 | <0,0001 | 0,0994 | 0,5793 | <0,0001 |
| CT 0.5h | <0,0001 | 0,1479 | 0,3279 | 0,3213 | 0,1402 | <0,0001 |
| CT 48h | 0,3438 | 0,0018 | 0,0005 | 0,0129 | 0,0097 | <0,0001 |
| UNT_CT1 | 0,1443 | 0,436 | 0,0023 | 0,674 | 0,1423 | 0,0262 |
| CT2 0.5h | <0,0001 | 0,1084 | 0,0178 | 0,6089 | 0,1947 | 0,3241 |
| CT2 48h | 0,5378 | 0,2595 | 0,0233 | 0,1846 | 0,0001 | 0,0054 |
| UNT_CT 2 | 0,0557 | 0,6986 | 0,0433 | 0,1619 | 0,0341 | 0,0278 |
| CT3 0.5h | <0,0001 | 0,9292 | 0,0368 | 0,0368 | 0,5565 | 0,157 |
| CT3 48h | 0,5136 | 0,1545 | 0,0035 | 0,1417 | <0,0001 | 0,0099 |
| 2Gy 0.5h | <0,0001 | <0,0001 | 0,0316 | 0,0022 | 0,0392 | 0,0666 |
| 2Gy 24h | 0,0116 | 0,0004 | <0,0001 | <0,0001 | <0,0001 | <0,0001 |
| 2Gy 48h | 0,0002 | 0,0062 | 0,0114 | 0,005 | 0,0014 | 0,0001 |

CT– 1st round of computed tomography, CT2– second subsequent diagnostic CT, CT3 – third subsequent diagnostic CT.
